# Friction and neuroimaging of active and passive tactile touch

**DOI:** 10.1038/s41598-023-40326-y

**Published:** 2023-08-11

**Authors:** Xue Zhou, Yiyuan Li, Yu Tian, Marc A. Masen, Yuanzhe Li, Zhongmin Jin

**Affiliations:** 1 State Key Laboratory of Tribology, Department of Mechanical Engineering, Tsinghua University, Beijing, 100084 People’s Republic of China; 2 Tribology Research Institute, Southwest Jiaotong University, Chengdu, Sichuan 610031 People’s Republic of China; 3 School of Economics and Management, Southwest Jiaotong University, Chengdu, 610031 People’s Republic of China; 4 Tribology Group, Department of Mechanical Engineering, Imperial College London, London, SW7 2AZ UK; 5 School of Mechanical Engineering, University of Leeds, Leeds, LS2 9JT UK

**Keywords:** Perception, Mechanical engineering

## Abstract

Two types of exploratory touch including active sliding and passive sliding are usually encountered in the daily life. The friction behavior of the human finger against the surface of objects is important in tactile perception. The neural mechanisms correlating to tribological behavior are not fully understood. This study investigated the tactile response of active and passive finger friction characterized with functional near-infrared spectroscopy (fNIRS). The friction test and fNIRS test were performed simultaneously using the tactile stimulus of polytetrafluoroethylene (PTFE) specimens. Results showed that the sliding modes did not obviously influence the friction property of skin. While three cortex regions were activated in the prefrontal cortex (PFC), showing a higher activation level of passive sliding. This revealed that the tribological performance was not a simple parameter to affect tactile perception, and the difference in cortical hemodynamic activity of active and passive touch was also recognised. The movement-related blood flow changes revealed the role of PFC in integrating tactile sensation although there was no estimation task on roughness perception.

## Introduction

Human beings often explore and perceive the external environment by touching with their fingers. During this process, the brain will actively modulate the finger motion parameters, such as the contact force, sliding direction, velocity, contact angle, etc., resulting in complicated tribological behavior. Meanwhile, the skin of the finger undergoes compressive and tensile deformation during the motion, which stimulates the mechanoreceptors and subsequently produces electrical signals transmitting to the cerebral cortex by neurons, producing the ‘feel’ to the object surface. Human skin is an active soft tissue with complex mechanical and physiological characteristics, and research into the biotribological mechanisms involved in tactile perception and the neural activity of brain have received significant attention in recent years^1,2^.

In daily life, two types of sliding modes are usually used by human beings when touching objects: active movement (participant stroking the surface) and passive movement (an imposed sliding of the surface against the finger). Active movements are accomplished spontaneously through the planning or execution of exercise, and passive movements are made by external forces^3,4^. There are several studies related to active touch and passive touch from a tribological point or a surface haptics point^5–7^. A similar finger vibrational behavior in both active and passive touch with textile fabrics was found^8^. By using an ultrasonic friction reduction device, passive touch without sliding may not provide perceivable frictional information^9^.

Different brain mechanisms are engaged during active and passive exploratory touch^10–12^, however the results of the previous studies were inconsistent to some extent. Active touch involves motor planning process. Individuals should make plan before making an activity. Once the action is generated, the brain would make further motor plans to adjust the action in response to the various sensory information induced by the activity^13,14^. In contrast, during passive activity, the brain can process the perceptual signals induced by actions, and the active adjustments are limited. Therefore, active motion might elicit greater and more distributed brain activity^15^.

Nevertheless, opposite results are not uncommon^10,12^. The sensation attenuation theory^16–19^ might provide an alternative interpretation. This theory focuses on discriminating between self- and externally-generated sensations. People have no difficulty to distinguish between the movements they make by themselves and the movements that are passively applied to their own body through the external forces. Attenuating self-generated tactile sensation depends upon a temporally-precise prediction of the sensory consequences of one’s actions. For example, tickling oneself produces a less intense sensation than being tickled by someone else, since people can predict the consequence of our own’s acts. Researchers^18,20^ argued that the purpose of sensory attenuation might be a means of enhancing the salience of unexpected external events.

In general, whether the passive touch elicits stronger and more widespread activation of brain areas than the active touch may depend on the tasks, meaning that the two processes compete to each other. Compared to the response to an unexpected outcome, there is more activation in active condition than in passive condition if there is more motor plan in the experimental task. Conversely, if a process of an unexpected outcome prevails in the experimental task, then the passive condition may stimulate greater brain activity. Thus, in the current study, the neural mechanisms relating the tribological properties of the finger skin to the tactile perception was investigated, we expected to design tasks with similar motion parameters in active and passive movements which were performed in the same environmental input.

Research from human and primate neurophysiology has demonstrated that prefrontal cortex (PFC), especially dorsolateral prefrontal cortex) (DLPFC), played a crucial role in this integrative process^21^, and the supplementary motor area (SMA) was considered critical in the motor planning, initiation and execution^22^ is involved in the performance of motor sequences^23^. Therefore, this work focused on the PFC region which is involved in the tactile information integration processes while individuals touch the surfaces of objects. Considering that the friction-induced skin deformation in active sliding was self-generated, while that in passive sliding which was externally generated may lead to an increase in the intensity of sensation. It is assumed that there would be a higher activation elicited by passive touch in the PFC, especially in DLPFC region which plays an important role in integrating sensory inputs, and no activation in SMA region suggesting the same motor planning occurred in both active and passive touch.

**Figure 1:**
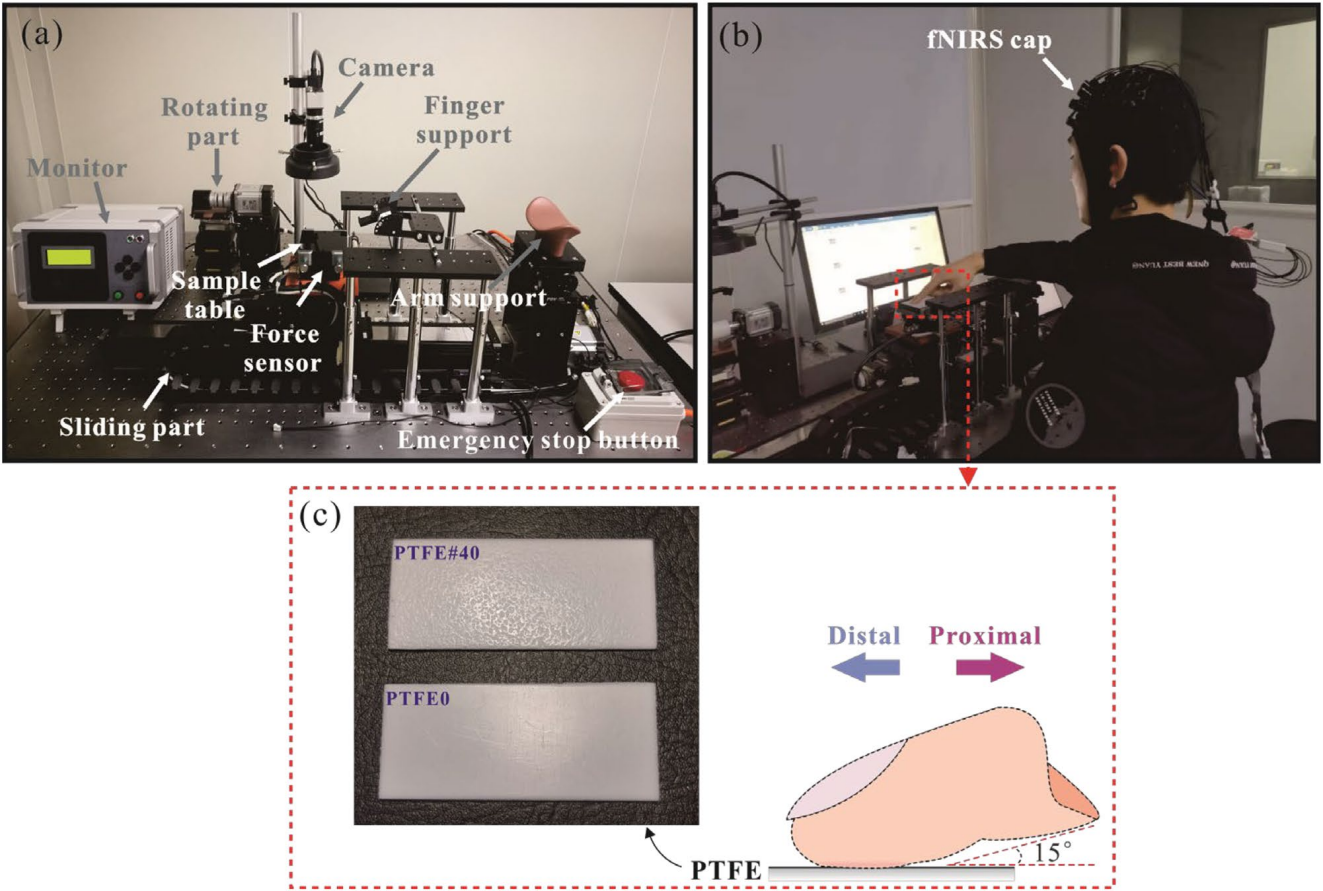
Custom friction experimental device (**a**), simultaneous measurement of friction test and fNIRS test (**b**) and schematic of finger-PTFE contact (**c**).

A novel measurement was carried out in this study, by combining the interface mechanics analysis and neuroimaging technique. The cortical hemodynamic activity in PFC were recorded by functional near infrared spectroscopy (fNIRS) imaging, simultaneously the friction between finger skin and PTFE sample were measured by using the custom experimental device, as shown in Fig. 1a. fNIRS is a non-invasive functional neuroimaging technique which uses near-infrared light to estimate cortical hemodynamic activity in response to neural activity^24^. The changes both in oxyhemoglobin (HbO) and deoxyhemoglobin (HbR) are mainly examined. Several previous studies investigated on human touch by using fNIRS^25–28^. Chen et al.^29^ investigated the tactile perception on fractal surfaces by using EEG-fNIRS study and found that comfortable sample activated the brain while uncomfortableness caused large entropy of EEG data.

By combining the friction measurement and fNIRS technology, this paper investigated the effect of active and passive touch on finger tactile friction behavior, assuming that there would be a higher activation elicited by passive touch in the PFC, especially in DLPFC region. From simultaneously recording mechanical input to brain output, this study could enrich the research method in mechanical and neuroimaging filed and provide guidance to the understanding of the tactile perception mechanism.

## Participants and methods

### Selection of participants

A total of 14 participants aged between 23 and 32 were recruited, with an average age of 26.2 years and SD of 2.78 years. They were postgraduate students from Southwest Jiaotong University, and were in good health without physical and psychological disorders in the last 6 months, with no injuries and scars on the index finger that was used in the research. Participants cleaned their fingers with hand sanitizer before commencing the experimental program. All measurements were performed in situ and were noninvasive at room temperature. This study was approved by the Ethics Committee of Southwest Jiaotong University (SWJTU-2205-QT (057)), and performed with the ethical standards of the Declaration of Helsinki. Informed consent obtained from all participants or their legal guardian(s) for publication of identifying information or images.

### Task design and procedures

Polytetrafluoroethylene (PTFE) specimens with dimensions of 30 mm × 70 mm × 1.5 mm were selected in this study. Two PTFE plates, noted as PTFE0 and PTFE#40 with increasing surface roughness were used. Figure 1(c) displays images of the PTFE samples, with S_q_ of 4.11 and 97.17 μm respectively. Detailed information on the specimens could be found in a previous study^30^.

**Figure 2:**
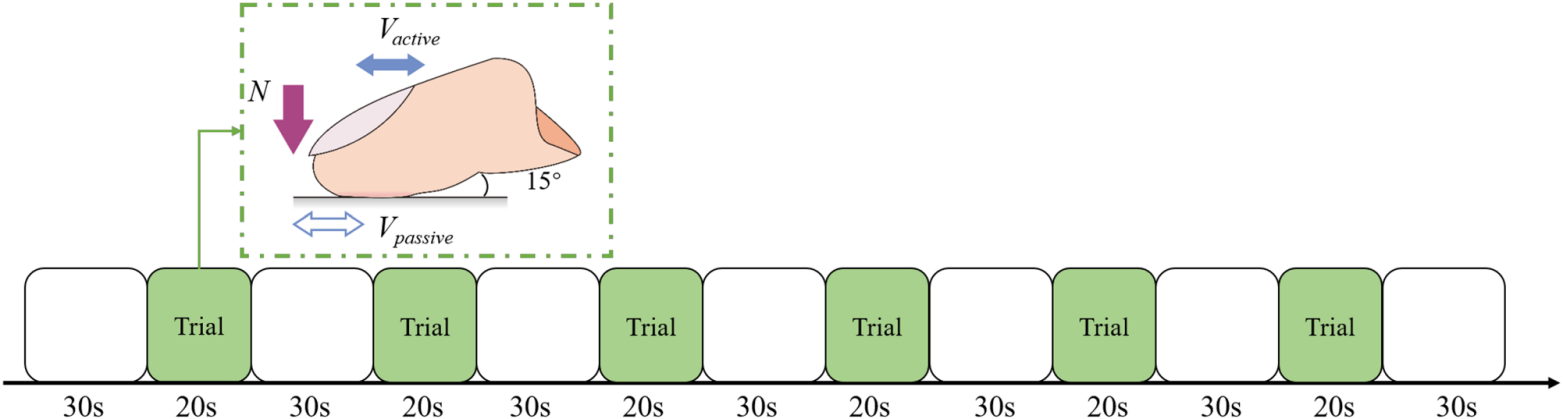
Schematic of experiment process.

The experiment adhered to a 2 (sliding modes: active friction vs. passive friction) × 2 (surface roughness: relatively smooth vs. relatively rough) within-subjects factorial design. The task design is illustrated in Fig. 2. There were 6 trials for each condition, meaning 24 trials for the whole experiment. Participants were instructed to put their index fingers on the PTFE specimens. In both the active and passive sliding conditions, they were asked to look at the monitor to maintain a normal force of approximately 1.5 N on the specimens throughout the movement. Finally, each participant rated their experience of sliding friction with the questions “Which sliding mode makes you feel it more strongly?” and “How similar is the tactile experience in the test to the daily experience when touching the surface of an object?”. The second question was assessed on a 9-point scale, the endpoints and midpoints defined as: not at all (1), and completely (9).

### Ethical approval

The study was approved by the Ethics Committee of Southwest Jiaotong University (SWJTU-2205-QT (057)). Participants provided written informed consent.

## Experiments

### Tribological test

The tribological test was performed simultaneously with the fNIRS test, as shown in Fig. 1b. The friction behavior involved in the fNIRS test was recorded using a custom setup with ‘finger down’ configuration, allowing finger active and passive reciprocated sliding. The setup comprised five main parts: the driving part, controlling the movement of a slider, the loading device including a force sensor, regulated by a lifting device, a finger and an arm holder with an angle sensor adjusting the position (Fig. 1a). The contact force applied on the finger by the slider was measured by a 3D force sensor with a measurement range of 100 N and a resolution of 5 mN. Detailed information of the device is available in the previous studies^31,32^. The PTFE specimens was glued onto the sample table mounted on the force sensor, and were cleaned by medical alcohol before each measurement.

In this study, two types of reciprocated sliding modes were analyzed, active motion and motion respectively. In active sliding mode, participants slid their index finger first in the proximal direction (toward the human body) for a distance of 60 mm, then slid in the distal direction (away from the body) for the same distance, forming one period of reciprocating motion. Participants were asked to performed three reciprocations in one trial. In passive sliding mode, the position of finger was kept static and the sample table was moved by the servo moto. During the sliding, the mechanical parameters, such as contact angle, sliding speed and normal force were set consistent, to ensure the same motion planning in both active and passive touch. Considering the touch habit of human used in the daily life^33–35^ and comfort of participants during tests, the contact angle between the fingertip and the PTFE sample was set as 15°, measured by a protractor. The sliding speed was set nominally as 24 mm/s. Participants should exert a normal force of 1.5 N on the sample by observing real time force signal on the computer. The measured force data were recorded using a data acquisition system (Jiangsu Donghua Testing Technology Ltd., China) with a sampling frequency of 2 kHz.

### fNIRS test

#### fNIRS device

**Figure 3:**
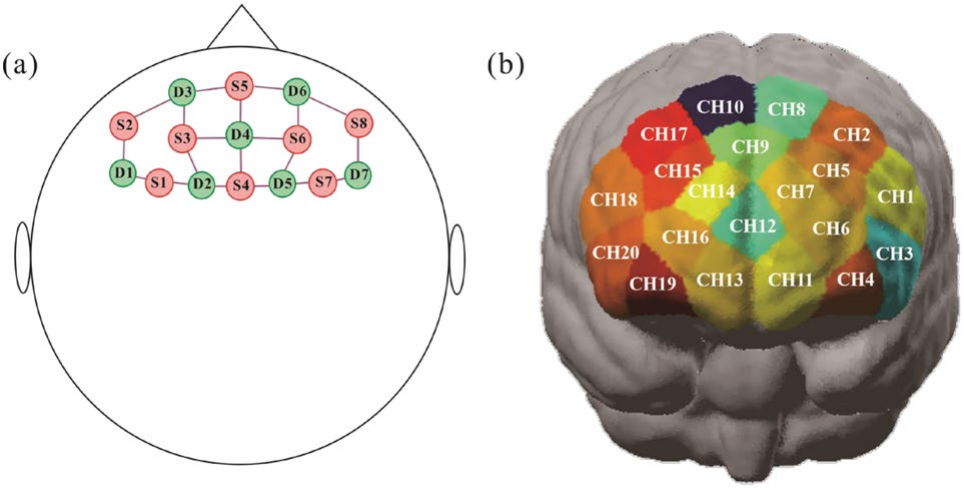
(**a**) Probe positions on 20 channels fNIRS cap, red dots: light source positions, green dots: detector positions. (**b**) 20 channels superimposed onto the PFC region of the head model.

A portable fNIRS device (NIRSport2, NIRx Medical Technologies, NY, USA) was employed to record hemodynamic information in participants' prefrontal cortex, measuring HbO, HbR and total hemoglobin (Hb). The device was equipped with 8 light sources (S1–S8) and 8 detectors (D1–D8), distributed on the fNIRS cap. The absorption of near infrared light at two wavelengths, 760 and 850 nm respectively were selected with a sampling rate of 10.2 Hz. In this study, 8 sources, 7 detectors and 20 channels were used for the PFC region. A channel was defined as the middle part of one source probe and one detector probe, a thin plastic straps were inserted between two types of probes to ensure that the distance was about 3 cm, benefiting the balance of the sensitivity and signal to noise ratio of the device. Figure 3 shows the distribution of source, detector probes on fNIRS cap and relevant channels (CH1–CH20) on the PFC region. Table R1 in Supplementary file shows the distribution of Brodmann area for the 20 channels.

**Table 1:** Result of questionnaire survey.

Participants	Which sliding mode gives the stronger feeling	Rating score for active sliding	Rating score for passive sliding
1	Passive sliding	8	3
2		9	2
3		8	6
4		7	5
5		8	3
6		7	5
7		8	7
8		8	2
9		8	5
10		6	3
11		7	5
12		8	6
13		8	3
14		7	3

In the fNIRS test, researchers assisted participants donning the fNIRS cap, the cap position should be centered at the midpoint of the head (Cz, indicating 50% of the distance between the nasion and the inion), and participants should remain calmed and relaxed. Every participant sat in front of the friction setup, without any large movement of the head and body during the experiment.

#### fNIRS data processing and analysis

The fNIRS raw data were processed and analysed by using the NirSpark software package on the Matlab 2014a platform (The Mathworks, USA). In the data process, the artifacts unrelated to the experimental data were removed; a band-pass filter of 0.01–0.2 Hz was selected to filter the noise and interference signals; the optical density converted into blood oxygen concentration based on the modified Beer-Lambert law. In this study, the HbO data were mainly investigated since this parameter was more sensitive to the regional cerebral blood flow. The processed HbO data were analysed by using the generalized linear model (GLM)^36^ and the hemodynamic response function (HRF, with − 2 s to 0 s as the reserved baseline state). The significance level for all analyses was set at 0.05 with FDR correction.

**Figure 4:**
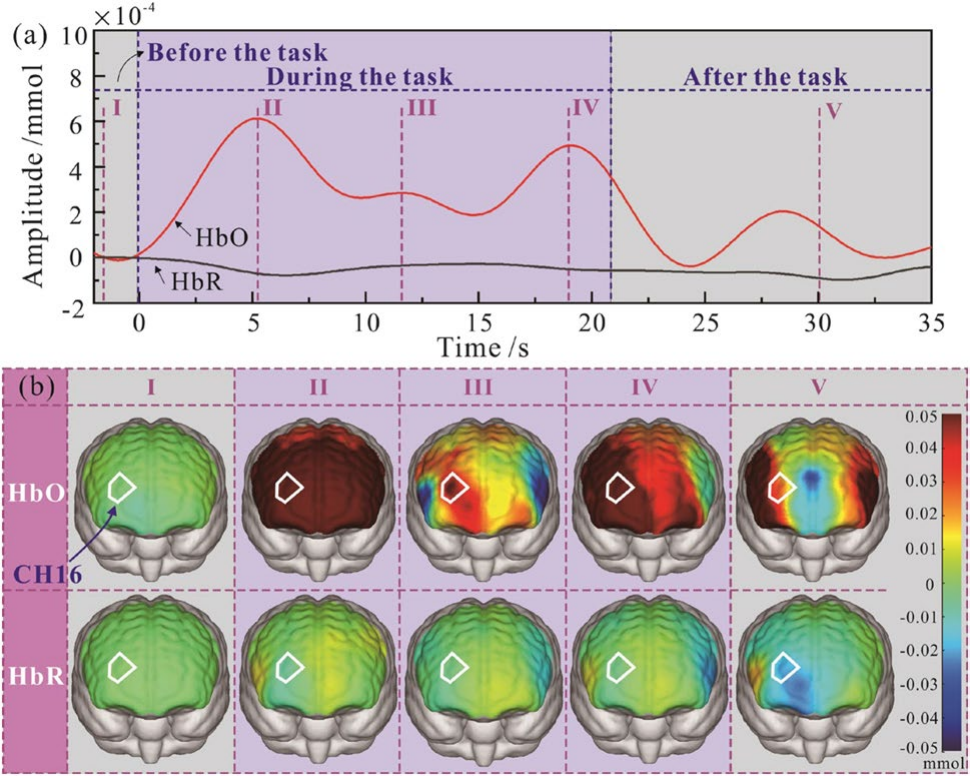
Schematic of fNIRS measurement collected in CH16, including the HbO and HbR signal (**a**) and the heatmap of HbO and HbR data obtained at 5 instants (**b**).

The Fig. 4a shows a typical fNIRS measurement collected in CH16, including the variation of HbO and HbR signal before and after a cognitive task. It can be observed that the HbO signal fluctuated significantly compared to the HbR signal. The HbO signal increased when the task started and subsequently decreased after the task, indicating the activation of the regional cerebral blood flow. By observing the heatmap of HbO and HbR obtained at 5 instants (I, II, III IV and V), as shown in Fig. 4b, a similar activation trend of the brain flow was found. The CH16 zone is circled by a white line. The state of HbR amplitude was stable and low, whilst the HbO varied significantly. There was no obvious activation of HbO before the task (at instant I). During the task (at instant II, III and IV), the zone turned red, the darker colour indicates the higher level of activation. The level of HbO activation varied with the time and reduced after the task (at instant V). Accordingly, the HbO was the sensitive parameter used to analyse the activation of the regional cerebral blood flow.

## Results

Each subject was able to perform the desired movement by using the index finger with reasonable accuracy. The data of all subjects were therefore included in the following analysis.

### Results of behavioral test

All subjects reported that the tactile stimulus deduced by passive mode was more intense than those by active mode, and the rated similarity of active sliding was significantly greater than passive sliding (*t*(13) = 7.16, *p* < 0.001, M_active_ = 7.64, SD = 0.74; M_passive_ = 4.14, SD = 1.61). Table 1 showed the rating scores for the similarity to the daily experience, produced by the active and passive sliding.

### Results of the friction test

**Figure 5:**
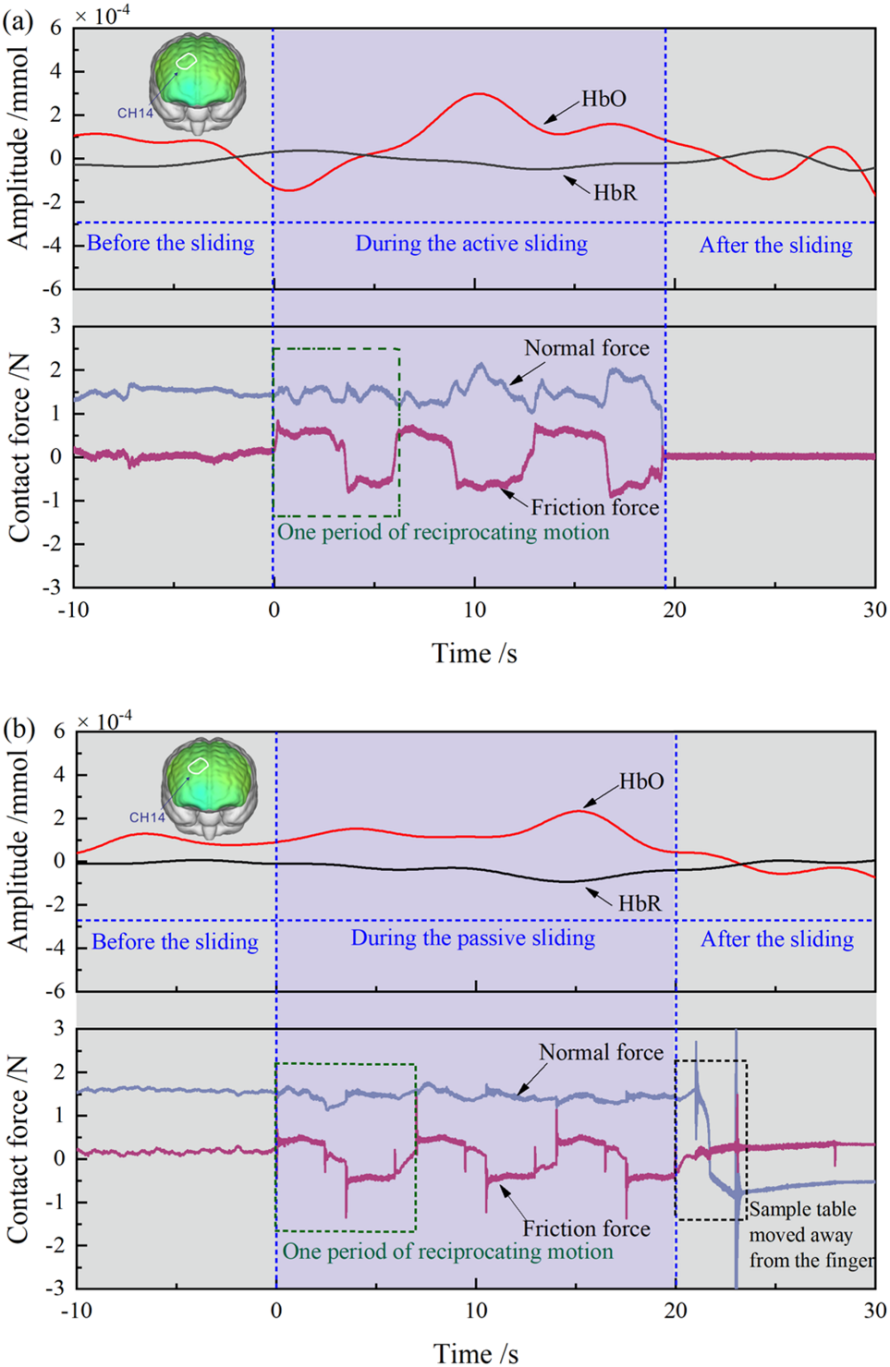
Comparison between fNIRS measurement and friction measurement for one trial, data obtained from a participant moving against PTFE#40 with active sliding mode (**a**) and passive sliding mode (**b**).

The Fig. 5 compares the fNIRS and friction measurements obtained from a single participant sliding against PTFE#40 for one trial. The changes of hemoglobin amplitude measured in CH14 are displayed in the graph, showing the relatively stable HbR signal and the fluctuant HbO signal. The friction measurement including the normal force and friction force were presented, comprising three periods of reciprocating motion. Participants were asked to keep the normal force at 1.5 N. It could be observed that the normal force measured in passive sliding (Fig. 5b) was more stable than in active sliding (Fig. 5a). Compared to the irregular amplitude variation of hemoglobin signal during the task, the friction force was rhythmical with the change of sliding direction.

**Figure 6:**
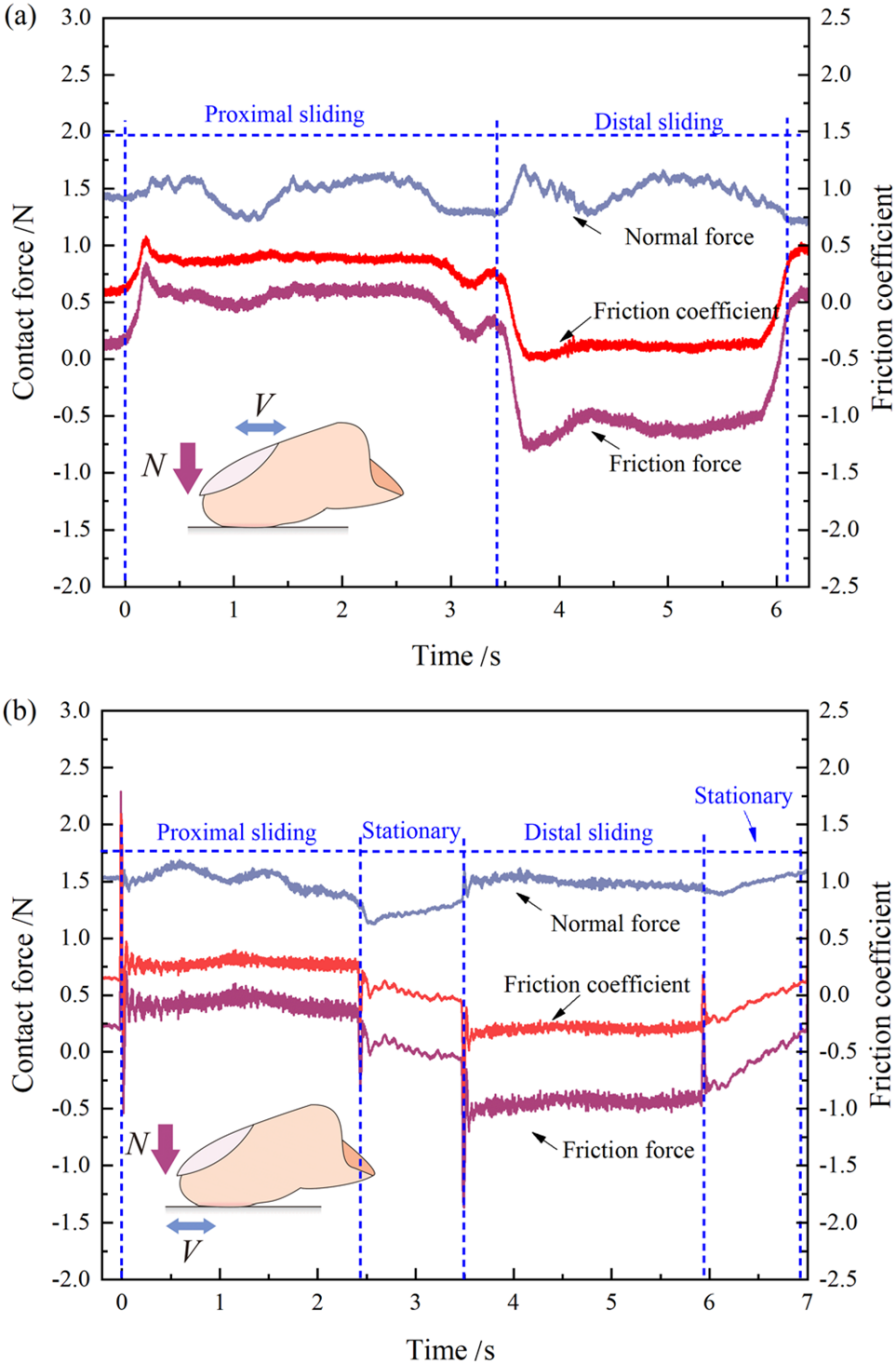
Typical friction measurement for one period of reciprocated sliding, including normal force, friction force and friction coefficients against PTFE#40: active sliding (**a**) and passive sliding (**b**).

The Fig. 6 displays a typical friction measurement for one period of reciprocation captured in Fig. 5 (green dashed area). During the test, participants slid their finger first in the proximal direction, then in the distal direction, performing the reciprocating motion, the normal force was controlled by participants through observing the real time signals. The graph shows the measured friction force, normal force and friction coefficient. It could be observed that the normal force was fairly constant at 1.5 N, the values of friction force and the friction coefficients were positive in the proximal direction and negative in the distal direction. In active sliding (Fig. 6a), participants would change the direction and move in the opposite direction immediately upon finishing in the proximal direction. In passive sliding (Fig. 6b), the motion of the sample table was controlled by the servo motor and there was a brief stationary state for the device to change the moving direction. The transition states were also shown in the contact force and friction coefficient signals.

**Figure 7:**
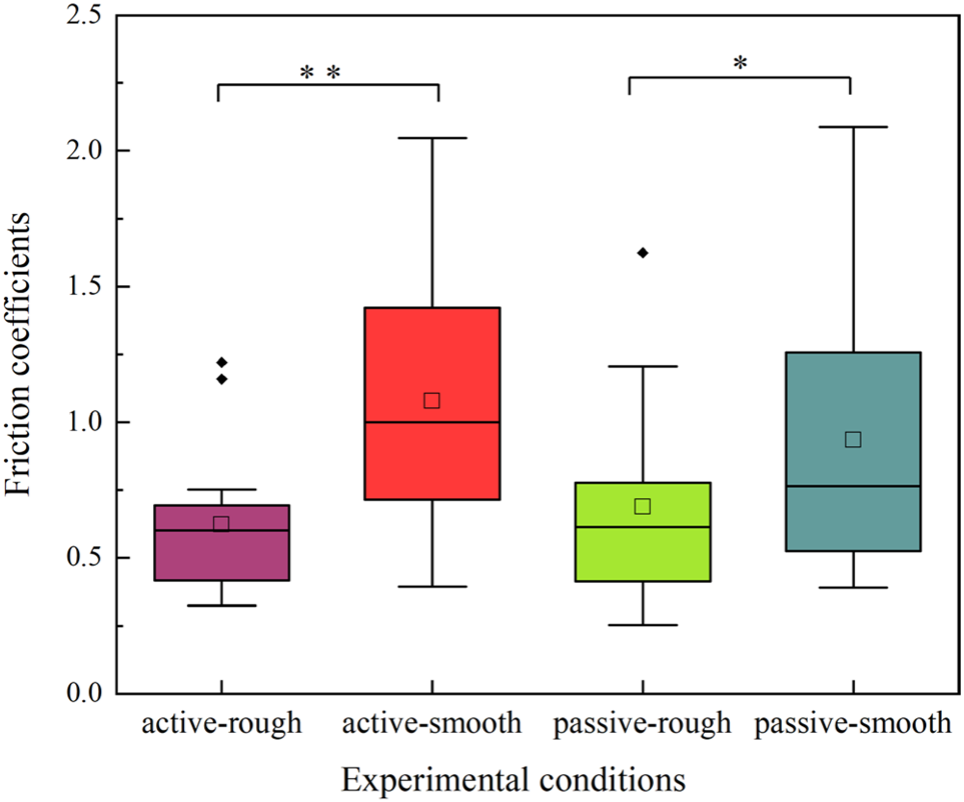
Friction coefficients measured under four experimental conditions, including two sliding modes and sample roughness. The “**” or “*” indicates a significant difference (*p* < 0.01 and *p* < 0.05 respectively) between two variables.

The distribution of friction coefficients from 14 participants measured under four experimental conditions are shown in the boxplots of Fig. 7. The average value of friction coefficients was calculated from the stable sliding region. In the boxplot, the size of the box indicates the first and third quartiles of sample data. The square marker represents the mean value and the horizontal line for the sample median. The error bar shows the total range of sample values, without outliers that are marked with small black points. In both two sliding modes, the distribution of friction coefficients measured on the rough sample was lower than that measured on the smooth one. For the same surface roughness sample, the distributions of friction coefficients were similar in active and passive sliding. By analyzing the effect of surface roughness, the paired sample t-test showed a significant difference both in active sliding (*t*(13) =  − 6.21, *p* < 0.01) and passive sliding (*t*(13) =  − 2.83, *p* < 0.05), indicating that the friction coefficient decreased with the increase of surface roughness. By analyzing the effect of sliding mode, there was no significant difference between the rough and smooth samples (*p* > 0.05).

### Results of fNIRS test

**Figure 8:**
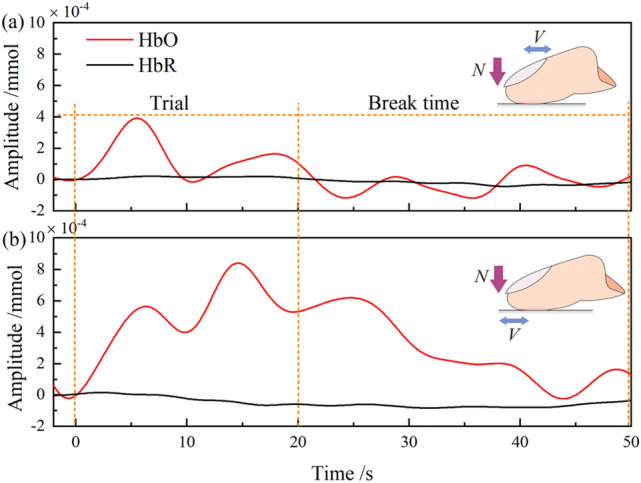
Measurement of HbO and HbR collected in CH7 for six trials from a participant moving against PTFE#40 with active sliding mode (**a**) and passive sliding mode (**b**).

The Fig. 8 shows the average measurement of HbO and HbR collected in CH7 for six trials from a participant moving against PTFE#40 with the two sliding modes. The sampling data were presented from − 2 to 50 s, the sliding task duration was 0–20 s as indicated by the dotted lines. It can be observed that during the task, the amplitude of HbR remained relatively stable while that of HbO fluctuated significantly. The amplitude of HbO increased obviously at the beginning of the sliding, then trended to decrease at the end of the task, meaning that HbO can reflect effectively the cerebral blood flow during the neural activity. In addition, the amplitude of HbO measured in passive sliding (Fig. 8b) was higher than in active sliding (Fig. 8a), indicating a higher level of activation occurring in CH7 with passive movement.

The HbO data from 14 participants were superimposed and analyzed statistically. Two regions of interest (ROIs) were analyzed by using 2 × 2 (sliding modes × surface roughness) repeated measures ANOVA. The first ROI was the pre-motor and supplementary motor cortex, including three channels, such as CH2, CH8 and CH10 as shown in Table R1 in Supplementary file. The ANOVA results shows that there was neither a significant main effect of the sliding modes, surface roughness nor were their relevant interactions (*p* > 0.05), indicating that there was no activation in the pre-motor and supplementary motor cortex. The second ROI was the dorsolateral prefrontal cortex, including CH7, CH14 and CH18. The ANOVA results revealed a main effect of the sliding modes (*F*(1,13) = 9.190, *p* < 0.05, *η*^2^_p_ = 0.231), and neither a significant main effect of the surface roughness nor were their relevant interactions (*p* > 0.05).

**Figure 9:**
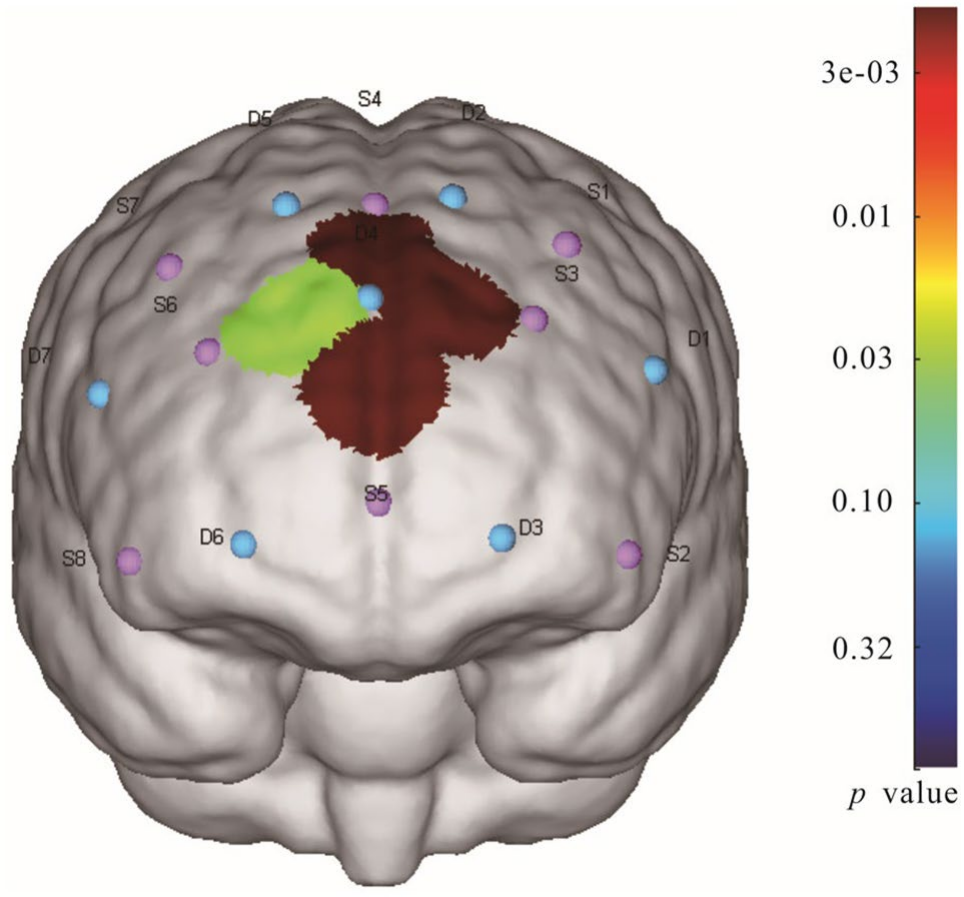
Statistically significant channels for HbO data between finger active and passive sliding modes on a head model.

To further investigate the effect of sliding modes in DLPFC, the paired sample t-test was employed between finger active and passive sliding, revealing a main effect of sliding modes occurred in CH7(*t*(27) = 4.392, *p* < 0.01), CH9(*t*(27) = 4.172, *p* < 0.01), CH12(*t*(27) = 4.141, *p* < 0.01) and CH14 (*t*(27) = 2.964, *p* < 0.05) while no significant difference in other channels (*p* > 0.05). The positive *t* value measured in activated channels indicates that a passive sliding stimulus produced more positive activation effects in relevant regions than an active stimulus, meaning a higher activation level for passive sliding mode, corresponding to the assumption. Figure 9 displays the activated channels between finger active and passive sliding modes on a head model, a highly significant difference occurred in CH7, CH9 and CH12. Table R1 in the supplementary file shows that CH7 includes the dorsolateral prefrontal cortex and frontal eye fields; CH9 is the frontal eye fields; CH12 is the frontopolar area; CH14 includes the dorsolateral prefrontal cortex, frontal eye fields and frontopolar area. Accordingly, three cortex regions, dorsolateral prefrontal cortex, frontopolar area and frontal eye fields were mainly activated with the two sliding modes.

## Discussion

A significant contribution of this study was the comparison of the neural basis underlying active and passive finger friction by controlling the external environmental input and task cognitive load. The friction test and the fNIRS test were performed simultaneously in the current study. The friction properties of finger skin showed significantly difference when sliding against samples with two surface roughness (*p* < 0.05). The friction coefficient decreased with increasing surface roughness. The main reason for this phenomenon was that within certain ranges, the real contact area of finger skin decreased with increasing sample roughness, resulting a reduction in adhesion between two surfaces^37^. Regardless of the surface roughness, similar friction coefficients were exhibited between fingers and surfaces whenever passively and actively moved. In other words, the employed sliding modes did not change the frictional properties of the skin in this work.

Although all participants maintained consistency in mechanical variables such as sliding velocity and normal force precisely in both two sliding modes, their self-reported experiences were still different: The passive movement produced higher stimulus intensities than the active movement, moreover, such experiences in passive sliding were more significantly different from daily experiences compared to those in the active mode. This result is consistent with the results of the previous studies^16–19^ that supported the sensory attenuation theory. Researchers^16^ provided a predictive explanation for one's inability to tickle oneself. That is, people have more accurate predictions of the consequences of self-produced actions, which leads to sensory attenuation, and conversely, people have more difficulty predicting the consequences of other-produced actions, therefore, have stronger feelings. The current study extends to one's perception of environmental objects. During the passive sliding, individuals also had more difficulty in accurately predicting the consequences of the action than the active sliding. Moreover, the results of behavioral test were also consistent with the corresponding brain activity in the current study.

The HbO was mainly investigated in the fNIRS test. It was found that different PFC regions were activated with different sliding modes during the task, the level of activation also varied. The first ROI analysis shows no activation in the pre-motor and supplementary motor cortex, which might be due to the similar motor planning in both active and passive touch since participants were required to maintain these mechanical parameters consistent in the two sliding modes. The second ROI approach revealed that a main effect of sliding modes occurred in the dorsolateral prefrontal cortex and the *T* test show the significant difference in CH7, CH9, CH12 and CH14, resulting in the activation of three cortex regions: dorsolateral prefrontal cortex, frontopolar area and frontal eye fields respectively. DLPFC is a key node of several brain networks, implicating in executive function, affective, and sensory processing, which can select task-relevant signals and efficiently suppress irrelevant stimuli^21,38^. In the current study, participants were asked to maintain the mechanical parameters consistent with two sliding modes, so they necessarily inhibited interference from task-irrelevant sensory stimuli during tactile processes. The greater activation in the passive sliding perhaps responds to the inhibition process of more intense feelings. This result also demonstrates that DLPFC is involved in the processing of tactile stimulation^39–41^.

The frontopolar cortex is a large region occupying the anterior portion of the brain’s frontal lobe, it has been suggested that the frontopolar cortex makes a crucial contribution to the exploration and rapid acquisition of novel behavioral options, which is an essential aspect of complex, higher order behavior^42^. Researchers^43^ considered that the frontopolar cortex was also related to the control of cognitive processes, but it does not act independently, rather augments or joins functionally with the dorsolateral prefrontal cortex.

The frontal eye fields involve the control of the eye movement, especially relates to the eye following movement. Participants in the present study had to watch the screen to maintain the mechanical parameters consistent, so this region was activated. Moreover, the difference between two sliding modes in activations might imply that there were stronger tactile stimuli involved in the process during passive sliding. This result is also self-consistent with the other results in this study.

The surface roughness estimation has been investigated in many psychophysical and neurophysiological research^15,44^. The different measured cortex region and perception task might lead to different conclusions under the two used sliding modes conditions. It was found a greater activation during active touch than passive touch in the contralateral primary somatosensory region^15^. By using fMRI, there was activation in the right prefrontal cortex during a roughness estimation task, compared with the no-estimation task, but little activation was observed during a no-estimation task in comparison with the resting phase^44^. The activation generated by roughness may be task-related. In this work, participants were not required to estimate the roughness of samples. The current fNIRS results shows that without estimation task, the PFC region was still activated. This indicates that the PFC region is essential in integrating tactile sensation even there is no roughness estimation task.

Friction test recorded the tribological behavior of finger sliding against PTFE samples, showing the mechanical properties of skin. The fNIRS test estimated cortical hemodynamic activity and measured the changes in oxyhemoglobin during finger tactile friction motion, reflecting the physiological and functional status of the brain. The compressive and tensile deformations of skin produced by the external mechanical stimulus stimulate the tactile center in the brain, inducing tactile sensations. A complicated procedure occurred from skin deformation to the brain response. The friction results show that there was no main effect of sliding modes on skin friction properties. However, the fNIRS results show a significant difference occurred on the change of regional cerebral blood flow. The difference between friction results and fNIRS results illustrate the complex neural activity process of the brain. The tribological performance is not a simple parameter to investigate the tactile perception mechanism, and the difference in cognitive behaviour may not be fully reflected in the tribological performance of the skin.

**Figure 10:**
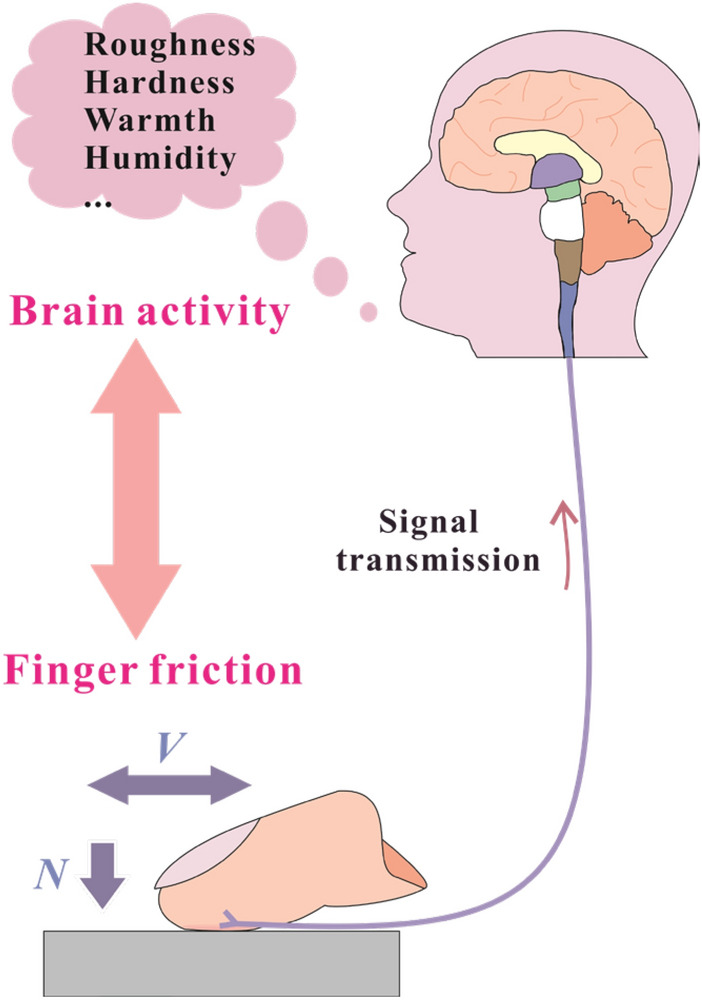
Schematic of finger tactile friction behavior.

There are some limitations to this study. Only PFC region was investigated and the fNIRS device was equipped with 8 light sources and 8 detectors, there was no more probes to further analyse the frontopolar area which occupied the largest area in PFC region. In addition, more participants (considering gender, age, profession etc.) could be invited in the friction-brain research. To further understand the human tactile sensation mechanism, the research of tribology and brain science should be correlated, as shown in Fig. 10. How to complement two disciplines to establish a quantitative relation between skin friction and perception should be paid more attention, for example, the effect of multiple mechanical parameters, such as contact angle, sliding speed and normal force on friction and brain signals should be considered. Furthermore, based on the current friction setup, developing a newly demagnetization technique to correlate MRI could perform wide mechanics-brain research. This will be helpful to understand the mechanism of tactile perception, the development of bionic skin and the fine touch design of intelligent robot^45–47^.

## Conclusion

Tactile response of finger and friction was investigated in active and passive sliding modes. An innovative research method was carried out, by simultaneously recording mechanical input to brain output. The tactile response was characterized by the changes of HbO and HbR in the PFC region, recorded with fNIRS technology, in conjunction with simultaneous tribological measurements by using a custom friction setup. This study could enrich the analysis method in the interdisciplinary neuroimaging- biotribology filed and provide guidance to the understanding of the tactile perception mechanism. The following conclusions can be drawn:

1. The friction coefficient was not markedly influenced by the sliding modes and decreased with increasing surface roughness of PTFE samples.
2. Different PFC regions were activated in two sliding modes, and the level of activation was also varied. The main effect was found to be the sliding modes, resulting in the activation of three cortex regions: dorsolateral prefrontal cortex, frontopolar area and frontal eye fields respectively. The activation level of passive sliding mode was higher than the active sliding mode. The movement-related change also revealed the role of PFC in integrating tactile sensation although there was no estimation task on roughness perception.
3. The tribological performance was not a simple parameter to affect the tactile perception, and the difference in cognitive behaviour may not be fully reflected in tribological performance. To further understand the human tactile sensation mechanism, the research of tribology and brain science should complement each other, from phenomenon to mechanism, single factor to multi-factor analysis, and organ level to cellular level.

### Supplementary Information

Supplementary Information.

## Data Availability

The data used to support the findings of this study are available from the corresponding author upon request.
