# Supplementary material for: Friction and neuroimaging of active and passive tactile touch
Source: Sci Rep. 2023 Aug 11;13:13077. doi: 10.1038/s41598-023-40326-y (PMC10421888; doi:10.1038/s41598-023-40326-y)
Supplement: Supplementary file 1 — Supplementary Information. [file 41598_2023_40326_MOESM1_ESM.docx]

**Supplementary file**

Table R1. Distribution of Brodmann area for 20 channels

| Channel | Probe | Brodmann area |
| --- | --- | --- |
| CH1 | S1-D1 | Frontal eye fields |
|  |  | Dorsolateral prefrontal cortex |
| CH2 | S1-D2 | Pre-Motor and Supplementary Motor Cortex |
|  |  | Frontal eye fields |
| CH3 | S2-D1 | Frontopolar area |
|  |  | Pars triangularis Broca's area |
|  |  | Dorsolateral prefrontal cortex |
|  |  | Inferior prefrontal gyrus |
| CH4 | S2-D3 | Frontopolar area |
|  |  | Orbitofrontal area |
| CH5 | S3-D2 | Frontal eye fields |
| CH6 | S3-D3 | Frontopolar area |
| CH7 | S3-D4 | Frontal eye fields |
|  |  | Dorsolateral prefrontal cortex |
| CH8 | S4-D2 | Pre-Motor and Supplementary Motor Cortex |
|  |  | Frontal eye fields |
| CH9 | S4-D4 | Frontal eye fields |
| CH10 | S4-D5 | Pre-Motor and Supplementary Motor Cortex |
|  |  | Frontal eye fields |
| CH11 | S5-D3 | Frontopolar area |
|  |  | Orbitofrontal area |
| CH12 | S5-D4 | Frontopolar area |
| CH13 | S5-D6 | Frontopolar area |
|  |  | Orbitofrontal area |
| CH14 | S6-D4 | Frontal eye fields |
|  |  | Dorsolateral prefrontal cortex |
|  |  | Frontopolar area |
| CH15 | S6-D5 | Frontal eye fields |
|  |  | Dorsolateral prefrontal cortex |
| CH16 | S6-D6 | Frontopolar area |
| CH17 | S7-D5 | Pre-Motor and Supplementary Motor Cortex |
|  |  | Frontal eye fields |
| CH18 | S7-D7 | Frontal eye fields |
|  |  | Dorsolateral prefrontal cortex |
| CH19 | S8-S6 | Frontopolar are |
|  |  | Orbitofrontal area |
| CH20 | S8-S7 | Frontopolar area |
|  |  | Pars triangularis Broca's area |
|  |  | Dorsolateral prefrontal cortex |
|  |  | Inferior prefrontal gyrus |
